# Benfotiamine Supplementation Increases Thiamine in Muscle of Endurance-Trained Mice and Affects the Energy Metabolism

**DOI:** 10.1155/2024/6102611

**Published:** 2024-09-26

**Authors:** Álisson C. Gonçalves, Jéssica F. Vieira, Ana Carolina N. Rodrigues, Eddie F. C. Murta, Júlio S. Marchini, Márcia A. Michelin, Guilherme V. Portari

**Affiliations:** ^1^ Department of Nutrition Federal Institute of Education, Science, and Technology Goiano, Campus Urutaí, Urutaí, GO, Brazil; ^2^ Oncology Research Institute Federal University of Triângulo Mineiro, Uberaba, MG, Brazil; ^3^ Experimental Nutrition Laboratory Federal University of Triângulo Mineiro, Uberaba, MG, Brazil; ^4^ Department of Medical Clinic University of São Paulo, Ribeirão Preto, SP, Brazil; ^5^ Department of Nutrition Federal University of Triângulo Mineiro, Uberaba, MG, Brazil

## Abstract

**Background:**

Benfotiamine, a synthetic analog of thiamine, offers greater bioavailability compared to other thiamine salts and increases thiamine stores upon oral intake. Thiamine is essential for energy metabolism. This study aimed to evaluate the effects of oral benfotiamine supplementation on energy metabolism, particularly the Krebs cycle function, in the muscle of endurance-trained mice, and to assess its impact on endurance performance.

**Methods:**

Twenty-five mice were randomly assigned to four groups: a standard diet with sedentary behavior (Sta-Sed), a benfotiamine-supplemented diet with sedentary behavior (Ben-Sed), a standard diet with swimming training (Sta-Tr), and a benfotiamine-supplemented diet with swimming training (Ben-Tr). The trained groups underwent five weekly swimming sessions for six weeks, followed by an exhaustive test. Thiamine and its esters were measured in erythrocytes and gastrocnemius muscle. Gene expression of pyruvate dehydrogenase (PDHa) and alpha-ketoglutarate dehydrogenase (OGDH), along with levels of pyruvic, lactic, and hydroxybutyric acids in muscle, was analyzed.

**Results:**

The benfotiamine-supplemented groups had higher thiamine levels in erythrocytes and muscles compared to the standard-diet groups. No differences were observed in PDHa and OGDH gene expression. The Ben-Tr group exhibited increased muscle lactic acid levels and a higher lactic acid to pyruvic acid ratio compared to the sedentary groups. Hydroxybutyric acid levels were also elevated in the Ben-Tr group. No significant differences in exhaustive test duration were found between the groups.

**Conclusion:**

Benfotiamine supplementation increases thiamine levels in erythrocytes and muscle but does not affect the gene expression of thiamine-dependent enzymes. Although it alters energy metabolism in trained muscle, it does not enhance endurance performance in mice.

## 1. Introduction

Physical exercise significantly increases the energy demands of cells, particularly in active muscle tissues. Thus, adequate nutrient intake is essential for optimizing energy metabolism and supporting tissue recovery [1]. While much research has focused on the impact of macronutrient intake on endurance performance, there is growing evidence that micronutrient deficiencies can also impair exercise performance. Moreover, supplementation with certain vitamins or minerals has the potential to enhance endurance capacity [2].

Thiamine, also known as vitamin B1, plays a critical role in energy metabolism. It exists in mammals in its free form or in three esterified forms: thiamine monophosphate (TMP), thiamine diphosphate (TDP), and thiamine triphosphate (TTP) [3, 4]. In the Krebs cycle, TDP acts as a coenzyme for the pyruvate dehydrogenase complex (PDH) and *α*-ketoglutarate dehydrogenase (KGDH) enzymes [5]. Thiamine deficiency is known to cause neurological disorders, such as beriberi and Wernicke–Korsakoff syndrome, which are associated with impaired energy metabolism in neuronal cells [6, 7].

Despite extensive research, there is no consensus on the effects of thiamine supplementation on exercise performance. Animal studies have shown that a thiamine-deficient diet decreases endurance capacity, but subsequent thiamine supplementation restores levels and improves endurance performance [8]. In humans, supplementation with thiamine derivatives has been suggested to have antifatigue effects and improve endurance [9]. However, research involving athletes supplemented with more absorbable thiamine analogs, such as allithiamine, has not demonstrated significant positive effects [10, 11].

Benfotiamine is a synthetic analog of vitamin B1 with greater bioavailability compared to other thiamine salts. It is converted to S-benzoyl-thiamine in the intestinal mucosa and is absorbed via passive diffusion, in contrast to thiamine salts, which require specific carriers to cross intestinal barriers [12, 13]. One study showed that oral benfotiamine supplementation increased TDP concentration in erythrocytes by 21 times compared to thiamine hydrochloride supplementation [12]. More recently, oral benfotiamine supplementation has been shown to enhance antioxidant defenses in the muscle and heart of endurance-trained mice [14].

Evidence indicates that benfotiamine can significantly elevate TDP levels in various tissues [15]. Given that TDP is a crucial cofactor in energy metabolism, this study hypothesizes that oral benfotiamine supplementation could increase TDP concentrations in muscle tissue, modulate the function of the energy production pathway, and thereby improve endurance performance in trained subjects. Therefore, the aim of this study is to assess the effects of oral benfotiamine supplementation on energy metabolism, particularly Krebs cycle function in muscle tissue, and its impact on endurance performance in trained mice.

## 2. Materials and Methods

### 2.1. Animals

Twenty-five 6-week-old male BALB/c mice were housed individually in plastic cages under an inverted 12/12 h dark cycle, at 22°C ± 1°C and 55% ± 5% humidity, with ad libitum access to feed (Table 1) and tap water. The mice were randomly assigned to four groups: the standard diet and sedentary group (Sta–Sed, *n* = 6), which received an AIN-93 standard rodent diet [16] and did not undergo training; the standard diet and trained group (Sta–Tr, *n* = 6), which received the AIN-93 diet and underwent endurance swimming training; the benfotiamine and sedentary group (Ben–Sed, *n* = 6), which received the AIN-93 diet supplemented with benfotiamine and did not train; and the benfotiamine and trained group (Ben–Tr, *n* = 7), which received the AIN-93 diet supplemented with benfotiamine and underwent swimming training.

The experimental protocol lasted 7 weeks. The first week was dedicated to acclimating the mice to the laboratory environment, the AIN-93-G standard diet, and their individual cages. The study was approved by the Ethics Committee of Animal Use at the Federal University of Triângulo Mineiro, under protocol number 343/2015.

### 2.2. Training Protocol

The animals swam in groups in a plastic container with a diameter of 40 cm and a height of 60 cm, filled with tap water to a depth of 40 cm, and maintained at 32°C (±1°C). The water temperature was controlled by an automatic thermostat heater (HOPAR SA-333, Zhong Shan, China) [17].

The first week of training served as an adaptation period, beginning with exposure to shallow water on the first day and culminating in a 60-minute swim on the fifth day. In the second week, the mice swam with an external metal load (1% of their body weight) attached to the proximal portion of the tail, for 60 minutes per session, 5 days a week. The load was increased to 2% of body weight over the following four weeks, with the duration and frequency of training sessions remaining consistent. The sedentary groups were exposed to shallow water with a similar frequency and duration as the training protocol used for the trained groups [17].

The final training session was designated for the exhaustive exercise test. Mice in the trained groups were subjected to an individual swimming session with an external metal load (5% of body weight) attached to the proximal portion of the tail. Exhaustion was determined when the animals were unable to keep their snout above the water surface for 8 seconds [17].

### 2.3. Determination of Lactate Concentration

Lactate concentration in peripheral blood was measured 5 minutes before and immediately after the exhaustive exercise test. A small incision was made in the distal portion of the tail, and 40 *μ*L of blood was collected using a heparinized tube. To the blood, 8.5 *μ*L of potassium fluoride/EDTA was added. The mixture was then centrifuged at 2000*g* for 5 minutes at 4°C. Subsequently, 10 *μ*L of plasma was used to analyze lactate concentration using a commercial kit (Intertek®, Brazil) according to the manufacturer's instructions.

### 2.4. Euthanasia and Tissue Preparation

The animals were euthanized by cardiac puncture exsanguination, following anesthesia with ketamine/xylazine [18], 24 hours after the exhaustive exercise test. Blood was collected in K3/EDTA tubes and immediately centrifuged at 3000*g* and 4°C for 5 minutes. The erythrocytes were washed three times with saline solution and then stored frozen at −20°C. The right gastrocnemius muscle was excised, washed in saline solution, and an aliquot (approximately 50 mg) of the medial portion was stored in RNAlater tissue storage reagent (Sigma-Aldrich®, Germany). The remaining muscle tissue was immediately frozen by immersion in liquid nitrogen. The gastrocnemius muscle was kept under liquid nitrogen until analysis.

### 2.5. Thiamine Quantification

The concentrations of thiamine, TMP, and TDP were analyzed in erythrocytes and gastrocnemius muscle. For erythrocyte analyses, the erythrocytes were hemolyzed in deionized water (1 : 1 v) by freezing and thawing three times with the aid of liquid nitrogen. Next, 0.8 M perchloric acid (1 : 1 volume) was added, and the mixture was centrifuged at 3000*g* for 10 minutes at 4°C to deproteinize. An aliquot of 80 *μ*L of the supernatant was then mixed with 50 *μ*L of 30 mM potassium hexacyanoferrate (K_3_Fe(CN)_6_) and 50 *μ*L of 0.8 M sodium hydroxide (NaOH) for derivatization. The solution was further mixed with 20 *μ*L of methanol, and 20 *μ*L of methanol was injected into a chromatograph (Shimadzu LC-10AT—Shimadzu Instruments, Japan). The analysis was performed using a mobile phase composed of 70 volumes of 25 mM phosphate buffer (pH 7.0), methanol, and acetonitrile (7 : 2 : 1 volume) with a C18 chromatographic column (Agilent, Sigma-Aldrich) and a flow rate of 1.0 mL/min. The fluorometric detector (Shimadzu RF-20A—Shimadzu Instruments) was set to 365 nm for excitation and 435 nm for emission [12]. The concentrations of thiamine and its esters (TMP and TDP) were normalized to the total hemoglobin concentration in the sample.

For the gastrocnemius muscle analysis, 50 mg of tissue was homogenized in 500 *μ*L of 50 mM sodium phosphate buffer (pH 7.0) and centrifuged at 3000*g* for 10 minutes. The total protein concentration was measured in the supernatant. An aliquot of the supernatant was then mixed with 0.8 M perchloric acid (1 : 1 v : v) and centrifuged at 4°C for 10 minutes for deproteinization. Subsequently, 200 *μ*L of the supernatant was mixed with 20 *μ*L of 30 mM potassium hexacyanoferrate diluted in 15% NaOH, and the solution was injected into the chromatograph system (Shimadzu LC-10AT—Shimadzu Instruments, Kyoto, Japan). The mobile phase consisted of 25 mM phosphate buffer (pH 7.0) and methanol (8 : 2 volume), and the stationary phase was an Ascentis RP-amide chromatographic column (Supelco, Germany). The fluorometric detector (Shimadzu RF-20A—Shimadzu Instruments, Japan) was set to 365 nm for excitation and 435 nm for emission [19]. The concentration of thiamine and its esters was normalized by the total protein concentration in the muscle supernatant.

### 2.6. Gene Expression Analyses

The expression of the E1-subunit alpha gene of the PDH complex (PDHa1) and the E1K component gene of KGDH (OGDH) was analyzed using qRT-PCR. Total RNA was extracted from 50 mg of gastrocnemius muscle (red portion), which was homogenized with 1 mL of TRIzol (Invitrogen, USA) according to the manufacturer's instructions. The total RNA was then converted to cDNA using the high-capacity cDNA-to-RNA kit (Applied Biosystems, USA), following the manufacturer's protocol. PCR was performed with 2 *μ*L of cDNA, 10 *μ*L of TaqMan® Fast Advanced Master Mix (Applied Biosystems, USA), 7 *μ*L of nuclease-free water, and 1 *μ*L of TaqMan® Gene Expression Assay for PDHa1 (Mm00468675_m1) and OGDH (Mm00803119_m1). RNA18s (Mm03928990_g1) was used as a constitutive gene. The reactions were performed using a 7900HT real-time PCR instrument (Thermo Fisher Scientific, USA), and gene expression levels were calculated using the ΔΔCT method, with data normalized to the Sta-Sed group [20].

### 2.7. Organic Acids Quantification

The quantification of pyruvic, lactic, and hydroxybutyric acids in an aliquot of the gastrocnemius muscle was performed using gas chromatography coupled with mass spectrometry (GCMS QP-2010 SE) (Shimadzu Instruments, Japan). Detection was conducted in both scanning mode (Scam) and selected ion monitoring mode (SIM) [21].

To achieve this, 500 *μ*L of distilled water was added to 50 mg of tissue, and the mixture was homogenized on ice using a Potter homogenizer. Then, 500 *μ*L of distilled water, 500 *μ*L of acetonitrile, and 0.2 *μ*g of an internal standard were added to 100 *μ*L of the homogenate. After vigorous vortexing for 2 minutes, the mixture was centrifuged at 20, 000*g* for 10 minutes to precipitate proteins. The supernatant was adjusted to pH 12 with 5.0 M NaOH. Following the addition of 1 mg of methoxyamine hydrochloride, the solution was incubated at 60°C for 30 minutes. The solution was then acidified to pH 1-2 with 10% sulfuric acid and saturated with sodium chloride. The analytes were extracted with 3 mL of diethyl ether and 2 mL of ethyl acetate. After adding 5 *μ*L of triethylamine, the combined extracts were evaporated to dryness under an argon gas flow at 40°C. The residue was resuspended in 20 *μ*L of toluene and 20 *μ*L of N-methyl-N-(tert-butyldimethylsilyl)trifluoroacetamide (MTBSTFA) + 1% tert-butyldimethylchlorosilane (TBDMS). The solution was heated at 60°C for 30 minutes, and the final solution was collected. Finally, 1 *μ*L of this solution was manually injected into a split-type injector [21].

### 2.8. Statistical Analysis

The data are presented as mean ± standard deviation. Results were compared, and graphs were created using GraphPad Prism software (Version 8.0.1). Levene's test and Shapiro–Wilk's test were employed to assess the equality of variances and data homogeneity, respectively. Two-way analysis of variance (ANOVA) was used to examine the effects of exercise training, supplementation, and the interaction between exercise training and supplementation on thiamine ester concentrations, gene expression, and organic acid concentrations. Nonparametric data, such as gene expression and organic acid concentrations, were log-normalized before performing the two-way ANOVA. Student's *t*-test was applied to compare exhaustive test times and plasma lactate concentrations. A significance level of 95% (*p* < 0.05) was adopted.

## 3. Results

**Figure 1:**
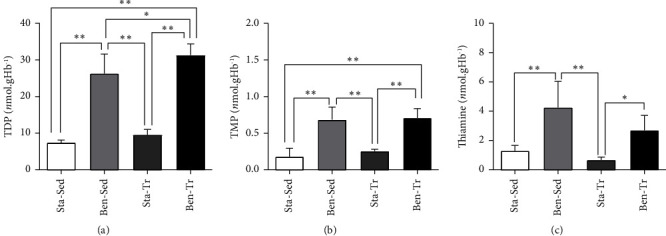
Concentration of thiamine and thiamine phosphates in the erythrocytes. (a) Thiamine diphosphate; (b) thiamine monophosphate; (c) free thiamine. ^∗^*p* < 0.05; ^∗∗^*p* < 0.01. Data are presented as the mean ± standard deviation (Sta–Sed *n* = 6; Sta–Tr *n* = 6; Ben–Sed *n* = 6; Ben–Tr *n* = 7).

**Figure 2:**
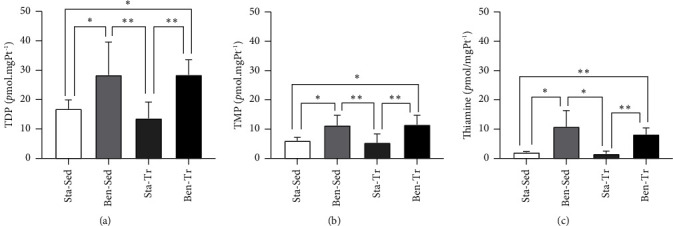
Concentration of thiamine and thiamine phosphates in the gastrocnemius muscle. (a) Thiamine diphosphate; (b) thiamine monophosphate; (c) free thiamine. ^∗^*p* < 0.05; ^∗∗^*p* < 0.01. Data are presented as the mean ± standard deviation (Sta–Sed *n* = 6; Sta–Tr *n* = 6; Ben–Sed *n* = 6; Ben–Tr *n* = 7).

There was no difference in benfotiamine intake between the supplemented groups (Ben-Sed and Ben-Tr). Supplemented animals exhibited higher levels of TDP in erythrocytes compared to the standard diet animals, both in erythrocytes (Figure 1(a)) and gastrocnemius muscle (Figure 2(a)). Notably, the Ben-Tr group had higher TDP levels in erythrocytes than the Ben-Sed group (Figure 1(a)). Two-way ANOVA analyses revealed a significant effect of supplementation on TDP (*p* < 0.0001), TMP (*p* < 0.0001), and free thiamine concentrations (*p* < 0.0001) in both muscle and erythrocytes. Additionally, for TDP and free thiamine concentrations in erythrocytes, the analyses also indicated an effect of exercise training (*p*=0.0108 and *p*=0.0166, respectively).

In both tissues, there was no significant difference in TDP concentration between the nonsupplemented groups. TMP levels in the erythrocytes and muscle of the supplemented groups were higher than those in the nonsupplemented groups (Figures 1(b) and 2(b)). Free thiamine concentration in the Ben-Sed group was higher than that in both nonsupplemented groups, while the Ben-Tr animals exhibited erythrocyte thiamine levels that were only higher than those in the Sta-Tr group (Figure 1(c)). However, free thiamine in the gastrocnemius muscle of the Ben-Tr group was higher than that in the nonsupplemented animals (Figure 2(c)).

**Figure 3:**
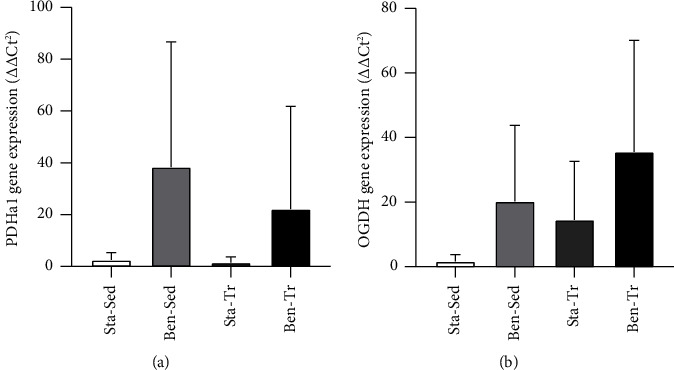
Target gene expression. (a) PDHa1 gene expression; (b) OGDH gene expression. Data are presented as the mean ± standard deviation (Sta–Sed *n* = 6; Sta–Tr *n* = 6; Ben–Sed *n* = 6; Ben–Tr *n* = 7).

There were no statistically differences in the expression of the PDHa1 and OGDH genes due to benfotiamine supplementation and/or training (Figure 3).

**Figure 4:**
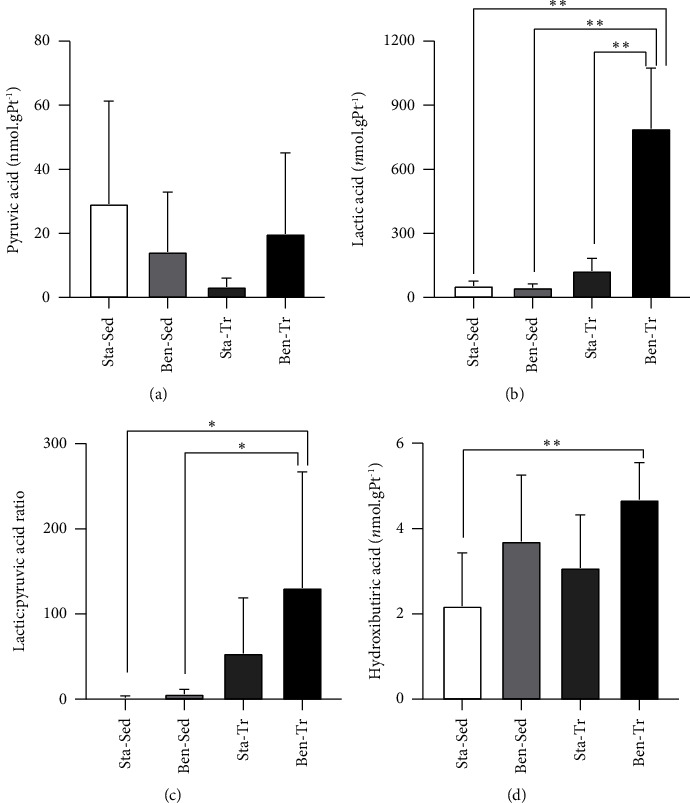
Concentration of organic acids produced in energy metabolism. (a) Concentration of pyruvic acid; (b) concentration of lactic acid; (c) lactic acid: pyruvic acid ratio; (d) hydroxybutyric acid. ^∗^*p* < 0.05; ^∗∗^*p* < 0.01. Data are presented as the mean ± standard deviation (Sta–Sed *n* = 6; Sta–Tr *n* = 6; Ben–Sed *n* = 6; Ben–Tr *n* = 7).

No significant differences were observed in pyruvate concentration in gastrocnemius muscle tissue (Figure 4(a)). In contrast, lactic acid concentration was significantly higher in the Ben-Tr group compared to both the Sta-Sed and Ben-Sed groups (Figure 4(b)), reflecting a notable effect of supplementation, exercise training, and their interaction. The lactic/pyruvic acid ratio was also significantly affected by the exercise training regimen, with the Ben-Tr group showing higher values than both sedentary groups (Figure 4(c)). Additionally, the Ben-Tr group exhibited increased levels of hydroxybutyric acid in muscle tissue compared to the Sta-Sed group (Figure 4(d)), underscoring the substantial impact of supplementation.

**Figure 5:**
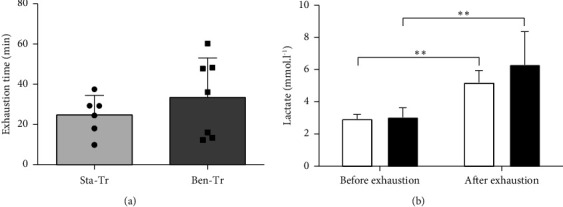
Endurance performance. (a) Exercise time until the exhaustion; (b) concentration of plasma lactate. White column: Sta-Tr group; black column: Ben-Tr group. ^∗^*p* < 0.05; ^∗∗^*p* < 0.01. Data are presented as the mean ± standard deviation (Sta–Tr *n* = 6; Ben–Tr *n* = 7).

The results of the performance in the exhaustive test revealed no significant differences among the groups. Furthermore, lactate concentrations did not differ between the groups; however, lactate levels were elevated postendurance test compared to pretest values in both groups (Figure 5).

## 4. Discussion

The objective of the present study was to investigate the impact of oral benfotiamine supplementation on components of energy metabolism associated with thiamine and its esters. Additionally, this study aimed to determine whether benfotiamine supplementation could influence the endurance performance of trained mice.

The results demonstrated that oral benfotiamine supplementation effectively increased free thiamine, TMP, and TDP in erythrocytes. It is well established that approximately 80% of TDP in whole blood is transported by erythrocytes. Despite the increase in thiamine phosphates in erythrocytes, this did not necessarily translate into increased TDP levels in tissues. Nonetheless, similar increases in thiamine phosphates were observed in the gastrocnemius muscle. Benfotiamine supplementation also led to elevated levels of TDP, TMP, and free thiamine in muscle tissue. These findings are consistent with other studies that reported increased concentrations of thiamine and its esters in erythrocytes, whole blood, neural tissues, and liver following benfotiamine supplementation [12, 15]. Notably, no studies were found that evaluated changes in thiamine ester concentrations in muscle tissue in response to benfotiamine supplementation. It is worth mentioning that the animals in the cited studies [12, 15] received benfotiamine via gavage, a method that can be stressful and invasive. In contrast, the present study administered the substance through dietary addition, which is a less invasive method of supplementation.

TDP, the biologically active form of thiamine, is a coenzyme of the PDH complex and the KGDH complex in the Krebs cycle [4]. The PDH complex acts to convert pyruvate molecules into acetyl-CoA, the precursor molecules of the Krebs cycle. KGDH catalyzes the conversion of *α*-ketoglutarate to succinil-CoA [22]. In both enzyme complexes, TDP acts in the component E1 [23, 24]. The gene expression of PDHa1 and OGDH, which are related to E1 components of the PDH and KGDH, respectively, was not different between the four groups, despite oral benfotiamine supplementation having increased approximately 3-fold the TDP levels in gastrocnemius muscle. Thus, an increase in TDP concentration seems not to affect the gene expression of the thiamine-dependent enzymes.

Few studies have been dedicated to investigating the role of thiamine supplementation in the gene expression of enzymes of the Krebs cycle. No study was found that investigated the gene expression of PDH or KGDH genes in response to thiamine supplementation. However, a study [25] showed that thiamine deficiency reduces mRNA levels of the enzyme transketolase and the component E1-subunit beta of the PDH, but no alteration was observed in PDHa1 and OGDH genes in three human cell types. The authors [25] suggest that thiamine does not affect the gene expression of all the components of thiamine-dependent enzymes. Another study [26] showed that there was no alteration in PDHa1 expression in human muscle after 1 and 8 weeks of endurance training. In accordance, the present study did not find any alteration in gene expression caused by exercise training.

Although benfotiamine positively affected the levels of TDP in muscle tissue, the concentration of pyruvic acid remained unchanged. The thiamine-dependent PDH complex is responsible for converting pyruvate molecules to acetyl-CoA, a substrate for the Krebs cycle [27]. Therefore, a decrease in pyruvic acid concentration in muscle tissue would be expected. However, endurance training also did not alter pyruvic acid concentration in the gastrocnemius muscle, despite exercise being known to increase pyruvate demand. This suggests that pyruvic acid concentration in muscle may not be influenced by pyruvate demand or increased PDH activity. Supporting this, a study [28] observed a significant increase in PDH activity without a corresponding decrease in intramuscular pyruvate concentration during isokinetic exercise.

In anaerobic glycolysis, pyruvic acid is converted to lactic acid by the enzyme lactate dehydrogenase [29]. It is important to note that the trained animals underwent an exhaustive exercise test, which significantly increases the utilization of anaerobic metabolism compared to sedentary or resting conditions. A study indicated that endurance cycling increases lactate concentration in human muscle [28]. In the present study, only the Ben-Tr group exhibited higher levels of intramuscular lactic acid. The two-way ANOVA analysis suggests both an isolated and a combined effect of endurance training and benfotiamine supplementation on muscle lactate metabolism. However, the results regarding the lactic/pyruvic acid ratio indicate that pyruvate-to-lactate conversion appears to be affected by training, but not by supplementation. The higher lactic/pyruvic acid ratio suggests that anaerobic metabolism was more heavily utilized in the trained animals.

Both glycolysis and *β*-oxidation can supply acetyl-CoA to the Krebs cycle. Acetyl-CoA molecules that do not enter the Krebs cycle are converted to ketoacids, such as hydroxybutyric acid [30, 31]. The Ben-Tr group exhibited elevated levels of intramuscular hydroxybutyric acid, suggesting an excess of acetyl-CoA. This may be due to the higher levels of TDP improving PDH activity, as observed in the brain and heart of mice [32]. Enhanced PDH complex activity increases the oxidative decarboxylation of pyruvate, promoting acetyl-CoA formation. Additionally, endurance exercise stimulates both glycolysis and fatty acid oxidation [33]. These factors could substantially raise acetyl-CoA levels, potentially exceeding the availability of oxaloacetate and thereby limiting the entry of acetyl-CoA into the Krebs cycle. Consequently, excess acetyl-CoA is converted to acetoacetate and subsequently to hydroxybutyrate [30, 31].

The elevated levels of lactate in the muscle of Ben-Tr animals may also result from increased acetyl-CoA production. Excess acetyl-CoA can inhibit PDH activity to prevent the formation of additional acetyl-CoA from pyruvate. Elevated acetyl-CoA levels stimulate pyruvate dehydrogenase kinase (PDK) activity, an enzyme that inhibits PDH [34]. Consequently, this inhibition of PDH leads to a greater conversion of pyruvate to lactate by lactate dehydrogenase.

The increase in plasma lactate concentration following the exhaustive exercise test, observed in both groups without significant differences between them, indicates that the animals likely reached similar levels of exertion, potentially leading to exhaustion. According to a study, thiamine deficiency can elevate lactate levels by reducing pyruvate dehydrogenase activity [35]. While TDP deficiency impairs PDH activity, an excess of TDP does not enhance enzyme activity to a degree that would affect the production/clearance ratio of serum lactate after exhaustive exercise.

Another study demonstrated that, in humans, 30 days of TDP infusion can reduce serum lactate concentration following a submaximal exercise test [36]. However, the rate of lactate production during an exhaustive exercise test is significantly higher than during a submaximal exercise, as anaerobic metabolism is highly activated during the final stages of exhaustive exercise [37]. Therefore, it appears that benfotiamine supplementation may not be sufficient to alter PDH activity to the extent required to reduce lactate production during maximal endurance exercise.

Benfotiamine supplementation did not improve the endurance capacity of trained mice. The results showed no enhancement in fatigue resistance in supplemented and trained animals compared to those that were trained but not supplemented. Evidence regarding the effects of thiamine supplementation on endurance performance is inconsistent. Several studies have reported that oral supplementation with various thiamine analogs can enhance exercise performance in both humans and animals [9–11, 38, 39]. However, no studies have specifically investigated the effects of benfotiamine supplementation on exercise performance.

Recently, a study demonstrated that TTFD supplementation increased exhaustive swimming time in male mice and also elevated glycogen levels in muscle and liver, which could enhance submaximal endurance performance [39]. Conversely, another study found that TTFD supplementation did not improve high-intensity exercise performance in humans [40]. Similarly, allithiamine, a thiamine derivative with high bioavailability, also failed to show an effect on endurance exercise performance in humans [10]. Additionally, intravenous TDP administration for 30 days increased maximal oxygen consumption (VO_2_max) in humans undergoing aerobic exercise [36]. Another study revealed that rats supplemented orally with dicethiamine hydrochloride (DCET) swam for a longer duration compared to those receiving thiamine hydrochloride or a placebo [41].

The antifatigue effects of thiamine and its analogs remain unclear in the scientific literature. Variability in thiamine derivatives used, administration routes, concentrations, and duration of protocols contribute to the lack of consensus on this issue. The present results indicate that the supplementation protocol employed does not constitute an effective antifatigue strategy. It is crucial to note that the effects on submaximal aerobic exercise performance may differ, as exhaustive endurance exercise relies significantly on anaerobic metabolism to sustain power output [37]. While aerobic metabolism is the primary source of adenosine triphosphate (ATP) during endurance exercise, performance in exhaustive exercise is constrained by anaerobic metabolism, which has a substantially lower capacity compared to aerobic metabolism [42].

Therefore, additional studies with higher levels of evidence are needed to clarify the effects of thiamine supplementation on exercise performance. Research involving a substantial number of human participants and exercise tests that accurately reflect endurance performance should be conducted to determine the actual necessity for a highly specific diet in thiamine and/or its derivatives. Furthermore, preclinical studies could enhance the understanding of the biomolecular pathways through which thiamine influences energy metabolism during exercise.

## 5. Conclusion

Oral benfotiamine supplementation increases free thiamine and thiamine phosphate forms in both erythrocytes and muscle tissue. Although benfotiamine supplementation does not impact the gene expression of component E1-alpha of pyruvate dehydrogenase or component E1 of the alpha-ketoglutarate dehydrogenase complex, higher TDP levels appear to be associated with changes in energy metabolism in the muscle of trained animals. However, this supplementation does not provide an antifatigue effect in endurance-trained mice subjected to an exhaustive exercise test.

## Figures and Tables

**Table 1:** Ingredients of AIN-93G diet (standard vs. supplemented).

Ingredients (g·kg^−1^)	Standard	Supplemented
Cornstarch	397.5	397.4

Casein (>85% protein)	200	200
Soybean oil	70	70
Dextrinized cornstarch	132	132
Sucrose	100	100
Fiber	50	50
Mineral mix	35	35
Vitamin mix	10	10
L-cystine	3	3
Choline bitartrate	2.5	2.5
Benfotiamine	0	0.1

Calories (kcal·kg^−1^)	3948	3948

## Data Availability

More details and information can be obtained in Alisson Gonçalves' thesis, available at the link: https://bdtd.uftm.edu.br/bitstream/tede/1054/5/TeseAlissonCGon%c3%a7alves.pdf. The data can be requested from the corresponding author at alisson.goncalves@ifgoiano.edu.br. The data belong to all the authors and will only be published and distributed with the consent of all the authors [43].
